# Integrin β3: structural functions, tumour microenvironment regulatory roles and targeted intervention strategies

**DOI:** 10.3389/fimmu.2026.1804949

**Published:** 2026-03-26

**Authors:** Lianyi Peng, Fuxian Liu, Yue Yang, Liying Zhang, Shuangsheng Huang, Fei Zhao, Caili Li, Jiyu Huang, Zihan Wang, Jianxin He

**Affiliations:** 1 Key Laboratory of Environmental Ecology and Population Health in Northwest Minority Areas, State Ethnic Affairs Commission, Northwest Minzu University, Lanzhou, Gansu, China; 2 Yunnan University of Traditional Chinese Medicine, Kunming, China; 3 Key Laboratory of Ministry of Education for Dunhuang Medicine and Transformation, Gansu University of Chinese Medicine, Lanzhou, Gansu, China

**Keywords:** angiogenesis, integrin β3 (ITGB3), targeted therapy, traditional Chinese medicine, tumour microenvironment

## Abstract

Integrin β3 (ITGB3) functions as a pivotal transmembrane receptor mediating bidirectional signalling between cells and the extracellular matrix within the tumour microenvironment (TME). Dysregulated ITGB3 expression activates downstream pathways such as FAK/PI3K-Akt/mTOR, orchestrating core malignant processes including invasion, metastasis, angiogenesis, immune evasion, and autophagy modulation. Beyond its mechanistic roles, ITGB3 serves as a valuable biomarker for early diagnosis and prognostic assessment. Therapeutic strategies targeting ITGB3 encompass small-molecule inhibitors, monoclonal antibodies, and emerging Traditional Chinese Medicine (TCM) formulations, which offer unique multi-component regulatory advantages. This review systematically elucidates the structure-function relationship of ITGB3, its multidimensional regulatory mechanisms in tumour progression, and current targeted intervention strategies. Ultimately, we aim to provide theoretical insights for establishing ITGB3-guided precision medicine and integrated treatment systems.

## 1 Introduction

The initiation and progression of tumours represent a complex process involving the coordinated regulation of multiple genes and pathways. Among these, cell-extracellular matrix adhesion and the remodelling of the tumour microenvironment constitute pivotal mechanisms driving tumour invasion, metastasis, drug resistance, and immune evasion (1). As a crucial class of cell surface adhesion molecules, integrins profoundly engage in several core aspects of tumour progression by mediating bidirectional signalling between cells and their microenvironment (2). Integrin β3 (ITGB3), a pivotal member of the integrin family, is encoded by a gene located on human chromosome 17q21.32. By forming functionally distinct heterodimers with different α subunits (e.g., αvβ3, αIIbβ3), it serves as a central node regulating cell adhesion and signal transduction (3).

In recent years, with the deepening of research into the tumour microenvironment, the pro-tumour role of ITGB3 has gradually been revealed. Unlike its physiological regulatory function in normal tissues, ITGB3 exhibits abnormally high expression in various malignant tumours (4). While ITGB3 is overexpressed in many cancers (e.g., breast, lung, melanoma), the expression levels and prognostic value vary by tumor type. For instance, high ITGB3 expression is strongly associated with poor prognosis in breast and lung cancers, whereas its clinical significance may differ in other contexts (5). It not only directly drives epithelial-mesenchymal transition (EMT) in tumour cells, enhancing their invasive and metastatic capabilities, but also constructs a microenvironment conducive to tumour growth and progression by regulating vascular endothelial cell function, remodelling the extracellular matrix structure, modulating the balance of immune cell subsets, and interfering with autophagy processes (6, 7). More significantly, ITGB3 expression levels correlate closely with tumour staging, metastatic potential, and patient prognosis, though this relationship exhibits heterogeneity across tumour types, providing crucial clues for early tumour diagnosis and disease assessment (8).

Despite the continuous expansion of therapeutic options for tumours, metastasis and drug resistance remain major clinical bottlenecks. Given ITGB3’s pivotal regulatory role in tumour progression, intervention strategies targeting ITGB3 represent a potential avenue for overcoming these clinical challenges. Multiple targeted therapeutics are currently advancing through fundamental research or clinical exploration phases. Concurrently, traditional Chinese medicine’s distinctive advantages in modulating the ITGB3 signalling pathway are garnering increasing attention. Consequently, systematically elucidating ITGB3’s structure-function relationship and its regulatory mechanisms in tumour progression, while summarising its application value as a biomarker and progress in diverse targeted intervention strategies, holds significant importance for advancing ITGB3-related fundamental research towards clinical translation and establishing novel, precise, and efficient tumour treatment protocols.

## 2 Structure and function of ITGB3

**Figure 1:**
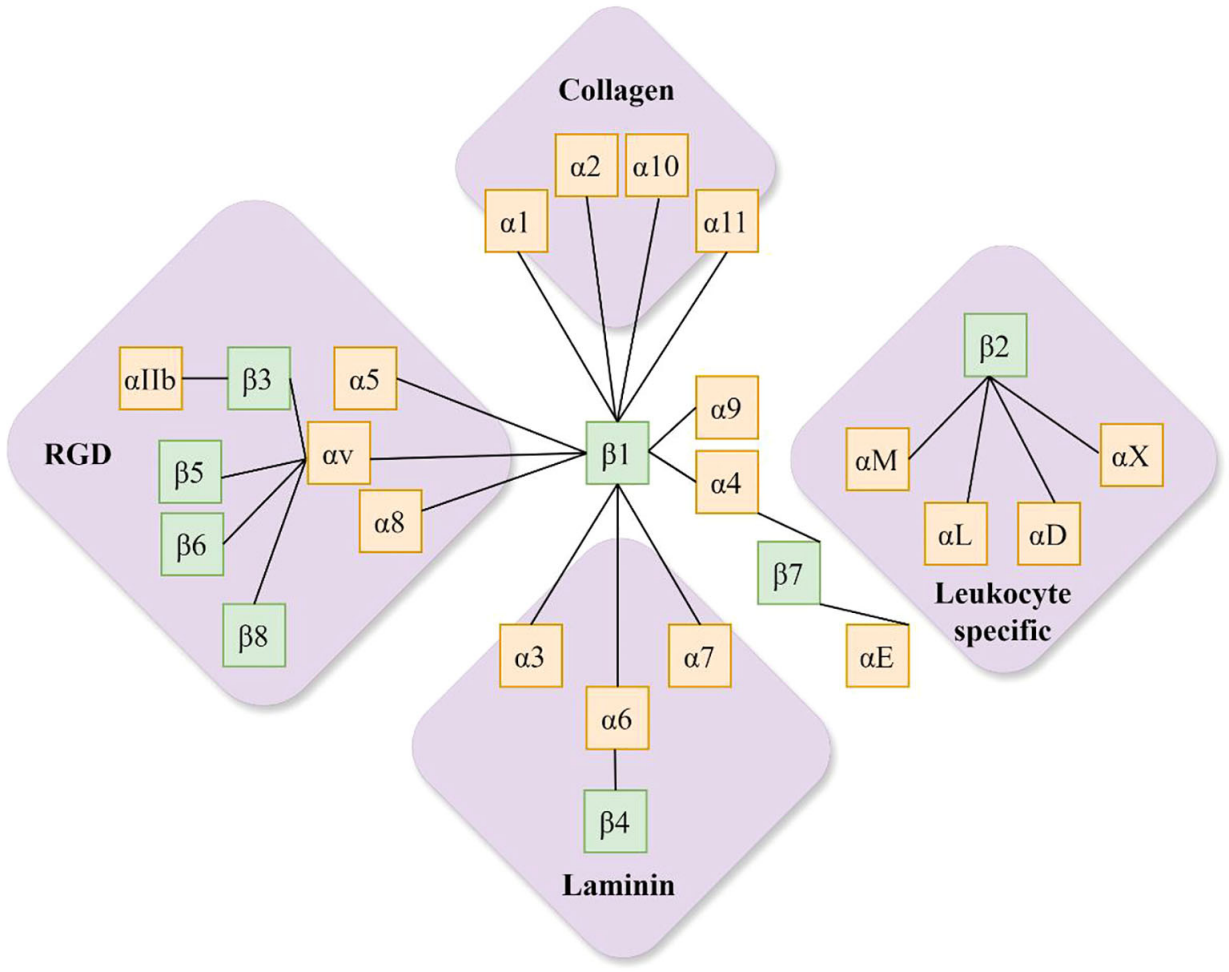
The integrin family (the β1 subunit can form heterodimers with 12 distinct α subunits, including α1, α2, α3, α4, α5, α6, α7, α8, α9, α10, α11, and αv. The β4, β5, β6, and β8 subunits each bind exclusively to one α subunit, forming α6β4, αvβ5, αvβ6, and αvβ8, respectively. The β2 subunit primarily pairs with αL, αM, αX, and αD to form leukocyte adhesion-associated integrins: αLβ2, αMβ_2_, αDβ_2_, and αXβ_2_. The β_3_ subunit pairs with αIIb (platelet-specific) and αv to form αIIbβ_3_ (involved in platelet aggregation) and αvβ_3_. The β_7_ subunit pairs with α_4_ and αE to form α_4_β_7_ (involved in lymphocyte homing) and αEβ_7_, each possessing specific ligands and functions.

Integrins are a crucial class of cell surface adhesion molecules, whose functional units comprise heterodimers formed by non-covalent bonds between an α subunit and a β subunit. The ITGB3 gene, located on human chromosome 17q21.32, encodes the integrin β3 subunit (ITGB3) (9). In mammals, the integrin family comprises 18 distinct α subunits and 8 β subunits, which can combine to form at least 24 different heterodimers. The β subunit can pair with different α subunits to form integrin receptors with distinct functions. For instance, ITGA2B encodes the αIIb subunit, while ITGAV encodes the αv subunit, both of which can bind to the β3 subunit. Specific pairing configurations are illustrated in **Figure 1**.

Structurally, integrins undergo a characteristic “switchblade” conformational change that regulates their affinity for ligands. In the resting state, integrins adopt a bent/closed conformation with low ligand affinity. Upon activation, they transition to an extended/open state, exposing the ligand-binding site (10). The ITGB3 subunit features a complex domain architecture, including the βI-like (or βA) domain, the Plexin-Semaphorin-Integrin (PSI) domain, and the hybrid domain. Ligand binding induces significant structural shifts, particularly a swing-out motion of the hybrid domain, which propagates conformational changes across the transmembrane region to the cytoplasmic tail (11, 12).

The functional specificity of ITGB3 is determined by its α partner, with a clear distinction between the two major heterodimers. The αvβ3 integrin is widely expressed in various cell types, including endothelial cells, smooth muscle cells, and numerous tumor cells, where it recognizes ECM components such as vitronectin and fibronectin. In contrast, αIIbβ3 is platelet-specific. Within platelets, the complex formed by ITGB3 and ITGA2B is termed GPIIb-IIIa (13, 14), which is crucial for platelet aggregation and participates in haemostasis and thrombosis. These receptors recognise and bind corresponding ligands within the extracellular matrix, thereby activating downstream signalling pathways that regulate diverse cellular behaviours.

**Figure 2:**
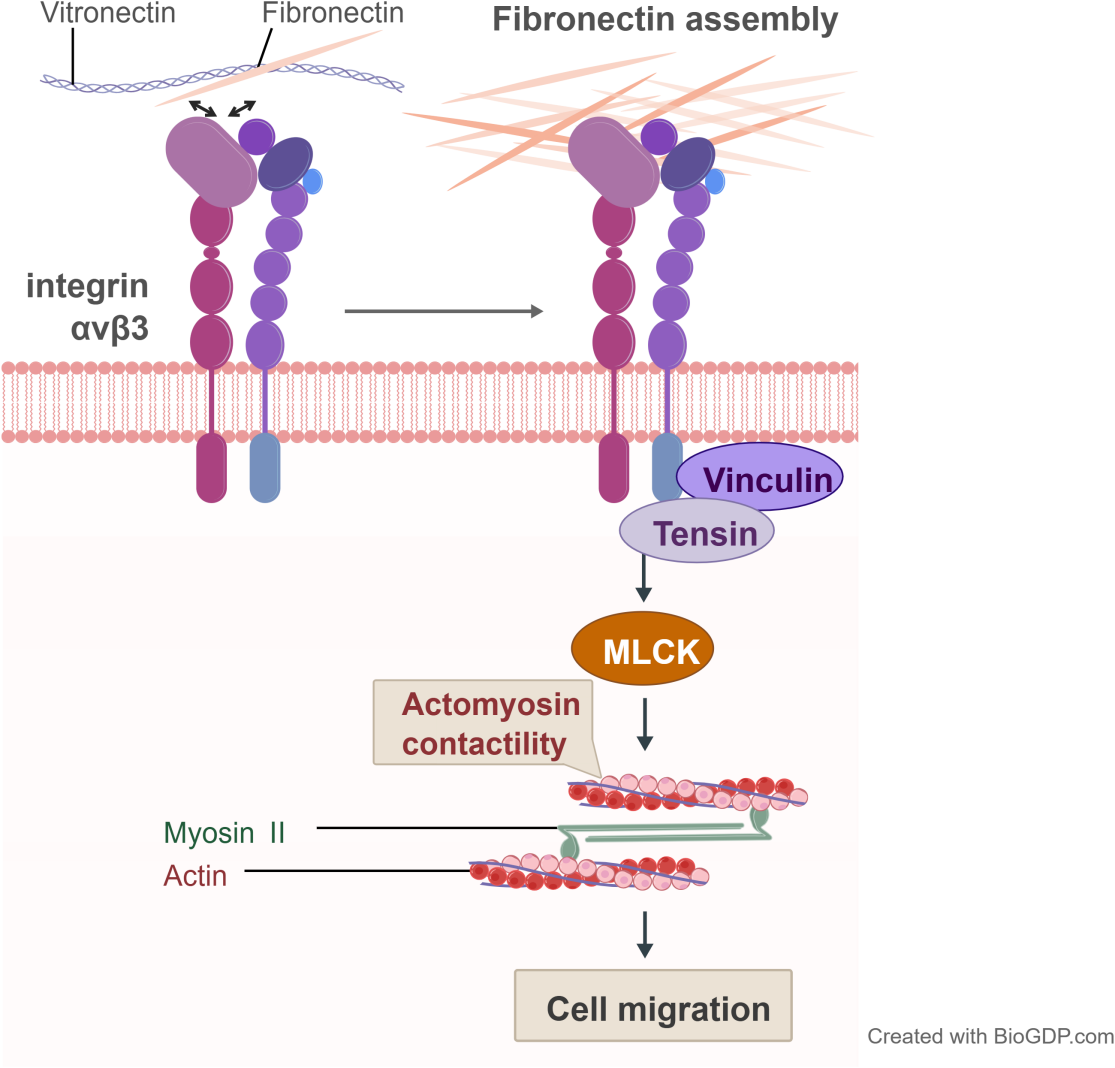
ECM-actin mechanical coupling mediated by integrin αvβ3 drives cell migration (integrin αvβ3 recognises vitronectin/fibronectin and transmits ECM tension via the vinculin-tensin-myosin II axis, driving actin-myosin contraction to facilitate cell migration).

Intracellularly, the cytoplasmic tails of ITGB3 play a critical role in signal transduction. They differentially recruit signalling molecules such as talin and kindlin, which are essential for inside-out activation by stabilizing the extended conformation. Conversely, ligand binding triggers outside-in signalling (15). This structural change facilitates the recruitment and activation of focal adhesion kinase (FAK) and Src family kinases (16). As illustrated in **Figure 2**, this ECM-actin mechanical coupling mediated by integrin αvβ3 drives cell migration (integrin αvβ3 recognises collagen VI/fibronectin and transmits ECM tension via the vinculin-tensin-myosin II axis, driving actin-myosin contraction to facilitate cell migration). Integrin β3 is a key member of the integrin family, playing a pivotal role in cell-extracellular matrix (ECM) interactions, cell migration, proliferation, and signal transduction (15).

To clarify the functional heterogeneity of ITGB3, we summarize the distinct roles of its two primary heterodimers in **Table 1**. While αvβ3 is the dominant form expressed on solid tumor cells and endothelial cells driving invasion and angiogenesis, αIIbβ3 is restricted to platelets. Despite not being expressed on tumor cells, αIIbβ3 plays a critical indirect role in metastasis by facilitating platelet-tumor cell aggregates that protect circulating tumor cells from immune surveillance.

**Table 1:** Functional specificity of ITGB3 heterodimers in tumor progression and microenvironment.

Heterodimer	Primary expression location	Dominant tumor types/contexts	Key ECM ligands	Main biological functions
αvβ3	Tumor cells;Endothelial cells;Osteoclasts;Smooth muscle cells	Solid Tumours: Breast, Lung, Melanoma, Glioblastoma, Colorectal Cancer. Angiogenesis: Tumor vasculature. Bone Metastasis: Osteolytic lesions.	Vitronectin Fibronectin Osteopontin Fibrinogen	Invasion & Metastasis: Mediates EMT, cell migration, and ECM degradation. Angiogenesis: Promotes endothelial cell survival and sprouting. Survival: Activates FAK/PI3K-Akt pathways to inhibit apoptosis. Immune Modulation: Regulates TGF-β activation and T cell infiltration.
αIIbβ3 (aka GPIIb-IIIa)	Platelets Megakaryocytes (Not expressed on tumor cells)	Metastasis: Circulating Tumor Cells (CTCs) interaction. Thrombosis: Cancer-associated thrombosis. All Metastatic Solid Tumours: Via platelet-tumor aggregates.	Fibrinogen Von Willebrand Factor (vWF) Fibronectin	Platelet Aggregation: Critical for haemostasis and thrombosis. Metastatic Seeding: Forms platelet-tumor aggregates to protect CTCs from immune surveillance and shear stress. Microenvironment: Releases growth factors (e.g., TGF-β, PDGF) to prime metastatic niches.

## 3 Multidimensional role of ITGB3 in tumourigenesis and progression

**Figure 3:**
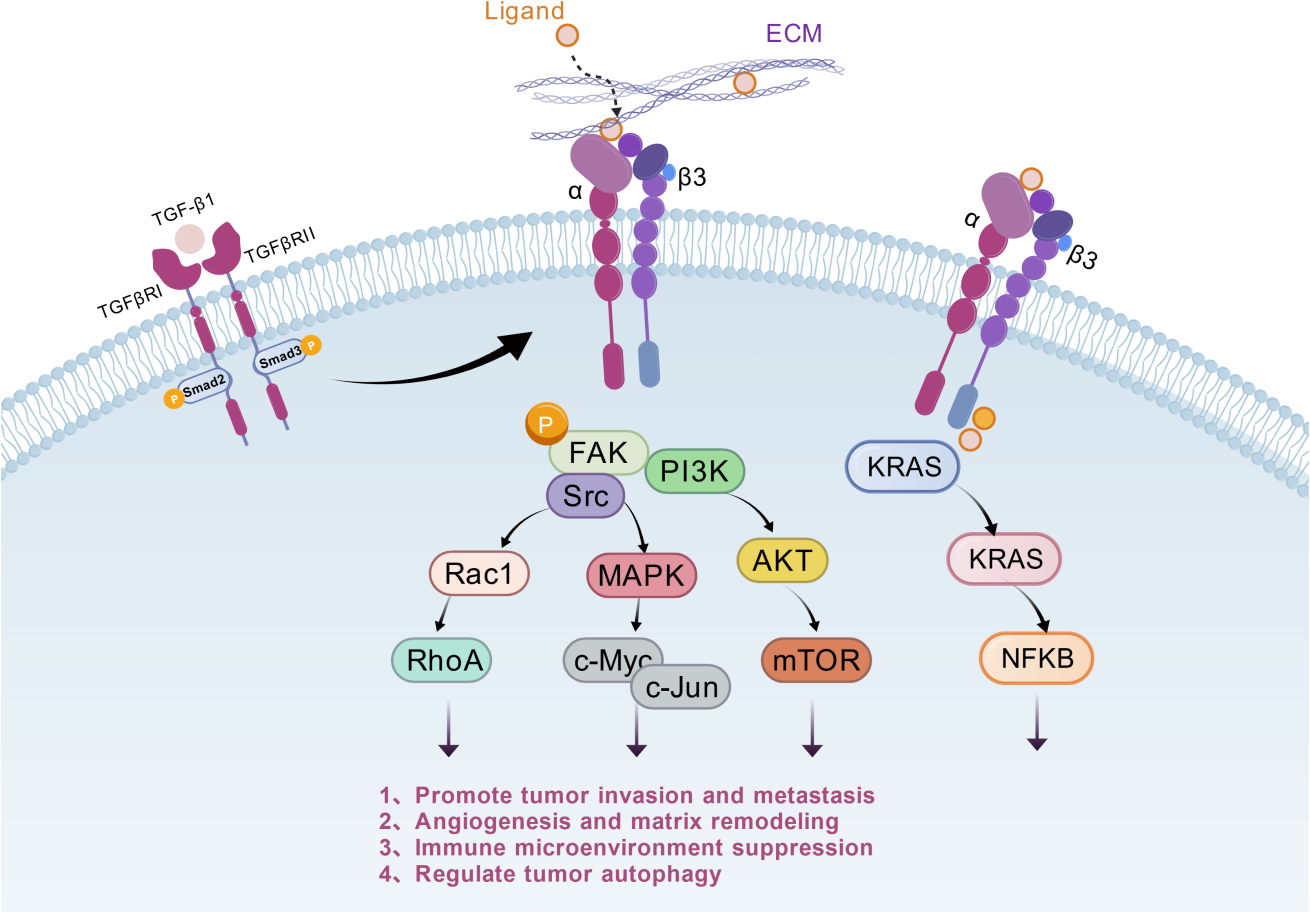
Regulatory role of ITGB3 in tumours (Following binding of extracellular ligands, extracellular matrix (ECM) components, or transforming growth factor-β1 (TGF-β1) to cell membrane receptors, ITGB3 activates FAK, Src, PI3K, KRAS, and other intracellular molecules. Via signalling pathways such as MAPK and AKT/mTOR, it ultimately regulates downstream molecules including c-Myc and NFκB, thereby promoting malignant phenotypes such as tumour invasion and metastasis, angiogenesis, immune suppression, and cellular proliferation.

ITGB3 exerts multidimensional core regulatory functions throughout the entire tumourigenesis and progression cycle. As a pivotal node in cell adhesion and signal transduction, it forms heterodimers with subunits such as αv and αIIb to mediate tumour cell adhesion to the extracellular matrix. This activates downstream pathways including FAK/PI3K-Akt/mTOR, driving tumour cell proliferation and inhibiting apoptosis, thereby laying the early tumor progression, survival, and establishment of the microenvironment (6). Concurrently, ITGB3 induces epithelial-mesenchymal transition, enhancing tumour cell invasion and migration capabilities to promote distant metastasis (14). By regulating vascular endothelial cell function, it accelerates tumour angiogenesis, providing nutritional support for tumour growth. Moreover, it remodels the tumour immune microenvironment, modulates the balance of immune cell subsets to facilitate tumour immune evasion, and optimises tumour cell survival by intervening in autophagy processes (4), as illustrated in **Figure 3**. The abnormal overexpression of ITGB3 is closely associated with tumour malignancy, metastatic potential, and poor prognosis, establishing it as a pivotal molecular hub linking tumour cells to the microenvironment and regulating malignant progression.

### 3.1 Promoting tumour invasion and metastasis

Epithelial-mesenchymal transition (EMT) and its concomitant invasion-metastasis cascade represent the most lethal stages in tumour progression, with integrin β3 (ITGB3) identified as a pivotal molecular switch in this process. In various models including breast, colorectal, and lung cancers, cytokines such as TGF-β1 and FGF1 released from the tumour microenvironment significantly upregulate ITGB3 expression (17), although this upregulation is context-dependent. For instance, while robustly validated in breast cancer models, the extent and mechanism of induction may vary in lung cancer contexts (18, 19). Subsequently, ITGB3 forms a complex with SRC family kinases via its cytoplasmic tail, leading to sustained activation of the SRC–FAK–MAPK/ERK axis. This sustained signalling is driven by conditions such as autocrine loops, matrix stiffness, or continuous ligand availability in the tumor microenvironment, rather than ligand-independent activation (20, 21). This pathway contributes to the phosphorylation and stabilisation of transcription factors Slug and Snail, often via intermediary kinases (e.g., through inhibition of GSK-3β) rather than direct phosphorylation by ERK in all contexts, thereby suppressing the expression of epithelial markers such as E-cadherin and tight junction protein ZO-1 (20). Concurrently, it induces the transcription of N-cadherin, vimentin, and matrix metalloproteinase-2/9 (MMP-2/9), conferring apical-basal polarity, contractile pseudopodia, and extracellular matrix degradation capabilities. This ultimately completes EMT and confers an invasive-metastatic phenotype (22). Galliher et al. (20) demonstrated for the first time using a mammary epithelial cell model that TGF-β1-induced EMT critically depends on β3 integrin. Knocking out ITGB3 blocked SRC kinase phosphorylation of the TβRII Tyr284 site, thereby inhibiting cross-activation of downstream p38 MAPK and NF-κB pathways. This prevented cells from downregulating E-cadherin, significantly impairing migration and invasive capacity. Reintroduction of ITGB3 restored both the signalling cascade and EMT phenotype, suggesting ITGB3 acts as the rate-limiting factor in TGF-β1’s functional switch from tumour suppression to promotion. Concurrently, Mori et al. (23) discovered that when FGF1 synergises with TGF-β, ITGB3 expression levels determine EMT efficiency: TGF-β alone induces only minimal EMT, whereas co-administration of FGF1 further upregulates ITGB3 via the ERK1/2-ETS1 axis, forming an “FGF1-ERK-ETS1-ITGB3” positive feedback loop. This significantly enhances Slug/MMP-9 expression and promotes basement membrane penetration. *In vivo* studies by Parvani et al. (24) established a triple-negative breast cancer orthotopic transplantation model, demonstrating that nanoparticle-mediated targeted delivery of ITGB3-siRNA reduced ITGB3 expression by 70% within lung metastases. accompanied by E-cadherin rebound and desmin reduction, with an 85% decrease in pulmonary surface metastatic nodules. This provided the first therapeutic evidence demonstrating that “silencing ITGB3 reverses EMT and blocks metastasis”. At the mechanistic level, ITGB3 not only functions as an adhesion receptor but also amplifies the EMT programme via bidirectional intracellular-extracellular signalling: its extracellular domain binds to matrix ligands such as fibronectin and osteopontin, inducing conformational changes in αvβ3 dimers that expose the NPLY motif at the β3 tail. This recruits and phosphorylates SRC at Tyr416. Activated SRC directly phosphorylates p130Cas and Dock180, thereby activating the Rac1-Arp2/3 pathway to promote lamellipodium formation. Concurrently, it translocates to the nucleus to phosphorylate Snail Ser246, enhancing its transcriptional suppression of E-cadherin expression (25). While supported by key studies in breast cancer, further validation across other tumor types is needed to confirm the breadth of this specific mechanism. Furthermore, ITGB3 physically interacts with TGF-β type II receptor (TβRII), reducing its ubiquitination and degradation, thereby prolonging TGF-β signalling duration. This forms an “ITGB3-TβRII-Smad2/3” positive feedback loop, further consolidating the EMT phenotype (26). Notably, ITGB3’s regulation of EMT extends beyond breast cancer. In lung cancer, the tobacco carcinogen NNK induces EMT via the αvβ3-SRC-NF-κB axis, whereas ITGB3 neutralising antibodies block NNK-induced invasion (27). In summary, ITGB3 promotes tumour tissue invasion and metastasis through multiple mechanisms. In-depth investigation of its mechanisms of action holds promise for identifying novel therapeutic targets and strategies to inhibit tumour metastasis.

### 3.2 Angiogenesis and matrix remodelling

A key step in tumour angiogenesis is the detachment, migration, and formation of a new capillary network by tumour vascular endothelial cells from pre-existing vessels (28). ITGB3 provides adhesive anchors for vascular endothelial cells by binding to ligands within the extracellular matrix (ECM), such as vitronectin, fibronectin, osteopontin, and fibrinogen. It significantly promotes tumour angiogenesis by activating downstream signalling pathways that drive cytoskeletal remodelling, thereby facilitating tumour growth, invasion, and metastasis (10).

Upon binding to its ligand, the intracellular segment of the β3 subunit of ITGB3 can interact with focal adhesion kinase (FAK), Src kinase, and integrin-linked kinase (ILK), forming a signalling complex (29). Phosphorylation of FAK (Tyr397) activates the downstream PI3K/AKT pathway, promoting vascular endothelial cell (VEC) survival; The FAK-Src complex activates Rho GTPases (e.g., Rac1, Cdc42), regulating actin polymerisation and pseudopodia formation to enhance cell migration. VEGF, a core driver of tumour angiogenesis, activates downstream PLCγ-PKC-MAPK/ERK and PI3K/AKT pathways by binding to VEGFR2 on VEC surfaces (30, 31). Research indicates that ITGB3 enhances VEGF sensitivity in VECs by promoting VEGFR2 endocytosis and recycling (e.g., via FAK-dependent pinocytosis) (32). Furthermore, ITGB3-activated FAK directly interacts with VEGFR2 to form an “integrin-receptor tyrosine kinase (RTK)” signalling axis, synergistically amplifying pro-migration signals (33, 34). ITGB3 signalling upregulates the expression and activity of proteases such as MMP-2 and MMP-9 (e.g., via the FAK-ERK pathway), degrading collagen and fibronectin within the basement membrane and ECM. This disrupts the physical barriers surrounding blood vessels, facilitating VEC infiltration into tumour tissue (35). During angiogenesis, ITGB3 is crucial for endothelial cell migration and luminal formation, with endothelial cell migration being a key step in tumour vasculogenesis.

Furthermore, ITGB3 can indirectly influence tumour angiogenesis by regulating the expression of angiogenesis-related factors. For instance, it upregulates the expression of vascular endothelial growth factor (VEGF) and its receptors. VEGF, a potent pro-angiogenic factor, exhibits increased expression that further promotes tumour vessel formation and maturation. ITGB3 facilitates tumour angiogenesis through multiple pathways, thereby providing essential nutritional and oxygen support for tumour growth and metastasis (36, 37).

### 3.3 Immune microenvironment suppression

ITGB3 functions as a critical immunomodulatory node within the tumour immune microenvironment (TIME), orchestrating immune evasion through a multi-layered mechanism involving immune cell differentiation, checkpoint regulation, and metabolic reprogramming.

Firstly, ITGB3 directly influences the differentiation and function of regulatory T cells (Tregs). ITGB3, particularly the αvβ3 heterodimer, is capable of activating latent TGF-β present in the extracellular matrix through mechanical force transmission. This activated TGF-β binds to receptors on naïve CD4^+^ T cells, triggering the Smad2/3 signalling pathway, which is essential for the transcriptional induction of Foxp3, the master regulator of Treg differentiation (38–40). Furthermore, ITGB3-mediated activation of the PI3K-Akt-mTOR pathway in tumour cells can lead to the secretion of cytokines (e.g., IL-10, TGF-β1) that further stabilise the Treg phenotype and suppress effector T cell function (41, 42). Pan-cancer analysis supports this (4), showing that ITGB3 overexpression correlates positively with Treg infiltration and negatively with CD8^+^ cytotoxic T cell activity, a signature associated with poor prognosis.

Secondly, there is a significant co-regulatory relationship between ITGB3 and immune checkpoint molecules. Emerging evidence suggests that ITGB3 signalling can transcriptionally upregulate immune checkpoint ligands. For instance, the ITGB3-FAK-Src axis has been shown to enhance the expression of PD-L1 on tumour cells via the PI3K-Akt and MAPK pathways, thereby facilitating immune escape (43). Conversely, immune checkpoint signalling can reinforce integrin activity; for example, PD-1 engagement may modulate integrin affinity states, promoting tumour cell adhesion and survival (44). This creates a positive feedback loop where ITGB3 signalling enhances checkpoint expression, and checkpoint engagement sustains integrin-mediated survival signals. Additionally, ITGB3 promotes the polarization of tumour-associated macrophages (TAMs) towards an M2-like immunosuppressive phenotype (45). Crucially, when ITGB3 is absent or blocked, this drive towards M2 polarisation is inhibited, thereby reducing immune evasion and restoring anti-tumour immunity.

Finally, ITGB3 contributes to metabolic-immune suppression. ITGB3 signalling elevates mitochondrial ROS output via the FAK-PI3K-AKT axis (46). This oxidative stress not only acts as a positive feedback loop for ITGB3 transcription but also induces apoptosis in cytotoxic T cells while attracting and stabilising Tregs. Neutrophil-derived reactive species (H_2_O_2_/HOCl) can further amplify ITGB3 expression, reinforcing the immunosuppressive niche (47).

In summary, ITGB3 acts as a “bidirectional immunoregulator” that shapes an immunodeficient microenvironment by depleting CD8^+^ T cells, expanding Tregs and M2 macrophages, and upregulating immune checkpoints. These findings provide a strong theoretical basis for combining ITGB3-targeted inhibitors with immunotherapy to overcome resistance.

### 3.4 Modulating tumour autophagy

Tumour autophagy can both suppress malignant transformation and provide metabolic buffering for tumour cells within hypoxic-nutrient-deprived microenvironments (48). Integrin β3 (ITGB3), a pivotal receptor for cell-matrix adhesion, has recently been identified as an ‘indirect modulator’ within autophagy regulatory networks. Three-dimensional culture experiments demonstrate (49) that when mammary epithelial cells lose adhesion to laminin, ITGB3-containing αvβ3 heterodimers undergo rapid internalisation and activate the IKK complex. This subsequently inhibits mTORC1 activity, inducing mTOR-independent autophagy to aid cellular resistance against apoptosis following loss of cell adhesion. Conversely, sustained overexpression of ITGB3 maintains mTOR signalling via the FAK-PI3K-AKT axis, reducing LC3-II lipidation levels to suppress excessive autophagy. This ensures cells retain an anabolic advantage post-detachment from their original location (50). Furthermore, pancreatic cancer exosome studies have defined ITGB4 as an “autophagy-associated exosomal gene”. Although both are integrin β subunits, ITGB4 possesses distinct cytoplasmic domains and signalling properties compared to ITGB3 (e.g., ITGB4 binds intermediate filaments, whereas ITGB3 binds actin); therefore, this mention highlights potential family-wide trends in exosomal regulation rather than structural identity. Its overexpression correlates positively with reduced autophagy activity, decreased CD8^+^ T cells, and increased M2 macrophages, suggesting that ITGB family members may suppress tumour cell autophagy via the exosomal pathway, thereby promoting invasion and immune evasion (51).

Although whether ITGB3 directly regulates LC3 lipidation or the ULK1 complex requires further validation, preliminary studies have revealed a novel link between “adhesion–autophagy–immunity”: ITGB3 dynamically coordinates mTOR and IKK signalling pathways according to extracellular matrix availability, thereby determining whether tumour cells initiate protective autophagy or shift towards metabolic reprogramming and metastasis. This discovery provides theoretical support for therapeutic strategies combining integrin inhibitors with autophagy modulators.

## 4 Prospects for targeted ITGB3 therapy in tumour treatment

### 4.1 As a biomarker for early tumour diagnosis

Research indicates that ITGB3 expression is significantly upregulated on the surface of various tumour cells, including those of breast cancer (52), and endometrial cancer (53). This characteristic renders the detection of ITGB3 expression levels a potential tool for early tumour diagnosis. Analysing ITGB3 levels through blood tests or tissue biopsies assists clinicians in detecting tumours at an earlier stage. Regarding prognostic assessment, ITGB3 enhances tumour cell adhesion, promotes metastatic invasion, and activates proliferative signals that drive tumour growth (8). Consequently, ITGB3 expression levels in patients serve as a crucial indicator for evaluating disease status and prognosis, aiding clinicians in formulating personalised treatment strategies.

Extensive research indicates that ITGB3 expression on the cell surface is significantly upregulated in many tumours during their early stages (54). In common cancers such as colorectal cancer, lung cancer, and multiple myeloma, ITGB3 levels in tumour tissue are markedly higher than in normal tissue. Currently, ITGB3 levels can be detected through multiple methods. Blood testing offers a convenient, non-invasive approach. Utilising immunoassay techniques such as enzyme-linked immunosorbent assay (ELISA), it enables precise measurement of circulating ITGB3 levels in the blood. Tissue biopsy, involving the acquisition of tumour tissue, provides a highly accurate method through immunohistochemical staining. This allows direct visualisation of the location and intensity of ITGB3 expression within cells.

Employing ITGB3 as an early tumour diagnostic marker offers dual advantages: firstly, it enables detection during the microscopic, asymptomatic stage of tumour development, significantly enhancing early diagnosis rates. Secondly, the testing process is relatively straightforward and rapid, with certain methods causing minimal patient discomfort and thus being readily accepted. However, both the specificity and sensitivity of detection require further refinement, and standardisation of testing protocols remains necessary (55).

### 4.2 Inhibitor screening and targeted drug therapy

ITGB3, as a key molecule in cell adhesion and signal transduction, plays a significant role in pathological processes such as thrombosis, tumour metastasis, and inflammatory responses. In colorectal adenocarcinoma (COAD), bevacizumab and regorafenib inhibit ITGB3-mediated angiogenesis by blocking VEGF. Inhibition of VEGFR/FGFR pathways blocks metastasis. Concurrently, bevacizumab, as an anti-VEGF monoclonal antibody, inhibits tumour angiogenesis and indirectly suppresses ITGB3 ligand activation, thereby reducing metastasis. Targeting PD-1 activates T-cell killing of uterine carcinosarcoma (UCS). Numerous tyrosine kinase inhibitors also downregulate ITGB3 expression, thereby influencing tumour cell development. For instance: sunitinib inhibits VEGFR and blocks mTOR, downregulating ITGB3 to modulate growth in renal papillary cell carcinoma (KIRP); axitinib targets ITGB3, highly expressed in renal clear cell carcinoma (KIRC), indicating strong vascular invasiveness, enabling anti-angiogenic therapy; Ibrutinib, a BTK inhibitor, suppresses the PI3K/AKT pathway, indirectly affecting tumour survival and migration; targeting PD-1 activates T-cell tumour killing, thereby regulating diffuse large B-cell lymphoma (DLBC) (56). Further inhibitor details are presented in **Table 2**.

**Table 2:** Therapies associated with ITGB3-related pathways.

Name	ITGB3	Mechanism	Medicinal products
Breast cancer	increase	ITGB3 and HER2 co-localisation promotes invasion; co-administration with celecoxib (a COX-2 inhibitor) blocks integrin activation.	Trastuzumab (52) Tozumab (57)
Lung cancer	increase	Inhibition of ITGB3-EGFR crosstalk signalling	Osimertinib (58)
Gastric cancer	increase	Inhibition of the ITGB3/PI3K pathway enhances chemotherapy sensitivity	Compound Kushen Injection (59)
Rectal cancer	increase	Targeting EGFR blocks downstream signalling, thereby indirectly inhibiting crosstalk between ITGB3 and EGFR.	Cetuximab (60)
Melanoma	increase	PD-1 inhibitors activate T cells; tumours with low ITGB3 expression exhibit higher CD8+ T cell infiltration.	Pembrolizumab (61)
Glioblastoma	increase	Targeting tumours with high expression	CAR-T (62)
Pancreatic cancer	increase	Inhibits VEGF-A-mediated angiogenesis, indirectly attenuating ITGB3-mediated endothelial cell adhesion and migration; downregulates the FAK/PI3K pathway.	Bevacizumab, Cabozantinib (63, 64)
Cholangiocarcinoma	increase	FGFR2 inhibitor, blocking FGFR2 fusion/rearrangement signalling	Pemetrexed (65)
Head and neck tumours	increase	Targeting EGFR blocks downstream signalling, indirectly inhibiting the ITGB3-mediated FAK/PI3K pathway, thereby reducing tumour invasion and angiogenesis.	Cetuximab (66)
Gastric adenocarcinoma	increase	Targets HER2, indirectly inhibiting crosstalk signals between ITGB3 and HER2; Small-molecule VEGFR2 inhibitor, suppressing the PI3K/AKT pathway.	Trastuzumab (67), Apatinib (68)
Low-grade glioma	increase	Inhibit MEK; target BRAF mutations	Simertinib (69), Tovorafenib (70)
Endometrial carcinoma	increase	Inhibit mTOR to downregulate ITGB3 metabolism; Block VEGF to suppress αvβ3 angiogenesis.	Everolimus (71), Bevacizumab (72)
Thyroid carcinoma	increase	Inhibit VEGFR, block ITGB3 angiogenesis	Selpatinib (73)
Renal clear cell carcinoma	increase	Inhibit VEGFR to block ITGB3-mediated angiogenesis; precisely target cells with high ITGB3 expression.	Sunitinib (74, 75)
Malignant melanoma of the skin	increase	Inhibit MAPK	cDabrafenib (76), Trametinib (77)
Bladder cancer	reduce	Improve the low-expression microenvironment of ITGB3; inhibit FGFR to indirectly regulate ITGB3 migration.	Atezolizumab (78)
Colonic adenocarcinoma	reduce	Inhibit VEGF to suppress ITGB3 angiogenesis; inhibit VEGFR/FGFR to block metastasis.	Bevacizumab, Regorafenib (79)
Pheochromocytoma	reduce	Inhibit VEGFR to block ITGB3 angiogenesis; inhibit mTOR to downregulate ITGB3 metabolism.	Pazopanib (80), everolimus (81)
Papillary carcinoma of the kidne	reduce	Inhibit VEGFR; block mTOR, downregulate ITGB3 metabolism	Sunitinib (82)
Renal clear cell carcinoma	reduce	High expression of ITGB3 indicates potent vascular invasiveness, warranting anti-angiogenic therapy.	Sunitinib/pembrolizumab plus axitinib (83, 84)
lung adenocarcinoma	reduce	Activate T-cell immunity to reverse the immunosuppressive microenvironment associated with ITGB3 hyperexpression (reducing Treg cells)	Nivolumab plus Ipilimumab (85, 86)
Lung squamous cell carcinoma	reduce	Activate T-cell immunity to reverse the immunosuppressive microenvironment associated with ITGB3 hyperexpression (reducing Treg cells)	Nivolumab plus Ipilimumab (87, 88)
Prostate adenocarcinoma	reduce	Blocking ITGB3 adhesion	Xilengitide + Abiraterone (89, 90)
Rectal adenocarcinoma	reduce	Targeting EGFR inhibits downstream MAPK/PI3K pathways, thereby affecting ITGB3-mediated cell adhesion and migration.	Cetuximab (91)
Endometrial carcinoma	reduce	Anti-angiogenic therapy to improve the tumour microenvironment	Bevacizumab plus chemotherapy (92, 93)
Uterine carcinosarcoma	reduce	Anti-VEGF monoclonal antibodies block tumour angiogenesis, indirectly inhibiting ITGB3 ligand activation to reduce tumour metastasis; targeting PD-1 activates T-cell tumour killing.	Pembrolizumab (94)
Tenosynovial giant cell tumour	reduce	Anti-angiogenesis combined with chemotherapy inhibits ITGB3-mediated drug resistance	Vemurafenib, Panitumumab (95, 96)
Diffuse large B-cell lymphoma	reduce	BTK inhibitors suppress the PI3K/AKT pathway, indirectly affecting tumour survival and migration; targeting PD-1 activates T-cell tumour killing.	Ibrutinib (97), Pembrolizumab (98)
Oesophageal cancer	reduce	Targeting HER2 for indirect regulation	Trastuzumab (99, 100)

### 4.3 Potential applications of traditional Chinese medicine and natural products targeting ITGB3

Numerous studies are increasingly focusing on the application of Traditional Chinese Medicine (TCM) and natural products targeting ITGB3 in tumour therapy. As a pivotal member of cell surface receptor families, ITGB3 regulates tumour cell proliferation, migration, and invasion. Consequently, targeted therapies against ITGB3 represent a promising strategy for cancer treatment. Unlike modern medical strategies that primarily focus on directly blocking ITGB3 function using monoclonal antibodies or small-molecule inhibitors, TCM employs a multi-component, multi-target approach to exert systemic regulation on ITGB3-centred signalling pathways. To improve clarity, this section is categorized into direct ITGB3-binding compounds, indirect modulators of ITGB3 signalling, and combination strategies.

#### 4.3.1 Direct ITGB3-binding compounds

Certain bioactive components within natural products have been demonstrated to act directly upon ITGB3, inhibiting tumour cell growth and metastasis. Zhang et al. identified an active compound extracted from a traditional Chinese medicine that specifically binds to ITGB3, thereby suppressing tumour cell proliferation and invasion (101). Similarly, research by Wang and Chen demonstrated that a novel ITGB3-targeted TCM exhibited favourable antitumour effects in both *in vitro* and *in vivo* experiments, with no apparent toxicity to normal cells (102). Liu et al. further discovered that a TCM formulation targeting ITGB3 significantly inhibited the proliferation and migration of breast cancer cells, offering novel therapeutic insights with broad clinical application prospects (62). These natural products not only possess potential antitumour effects but also provide novel leads for developing direct ITGB3-targeted therapies.

#### 4.3.2 Indirect modulators of ITGB3 signalling

Several TCM formulations and natural products regulate ITGB3 expression or downstream signalling pathways indirectly.

Qihuangzhuyu Formula (QHZYF): While primarily known for coronary artery disease, QHZYF’s mechanism has significant implications for tumour metastasis. Platelet activation (via αIIbβ3, a homolog of ITGB3) facilitates tumor metastasis by forming microthrombi that protect circulating tumor cells (CTCs). QHZYF mitigates this by inhibiting the PI3K/Akt/αIIbβ3 pathway, suppressing platelet membrane protein activation and inflammation (103). By inhibiting platelet adhesion and activation, QHZYF indirectly hinders tumor metastasis associated with microthrombus formation. Mechanism validation using PI3K and Akt inhibitors confirmed that QHZYF improves microvascular thrombosis primarily through inhibiting phosphorylation to suppress platelet membrane protein activation (103). Other Formulations and Compounds: Ma Huang Lian Qiao Chi Dou Tang (MHLQCD) protects hepatocytes from death via VWF/RAP1B/ITGB3 signalling, showing efficacy in liver failure models relevant to tumour microenvironments (104).

Specific Natural Products: Curcumin inhibits the proliferation and metastasis of triple-negative breast cancer (TNBC) cells by downregulating ITGB3 expression via the Hedgehog/Gli1 pathway, subsequently suppressing epithelial-mesenchymal transition (EMT) and breast cancer stem cell (BCSC) characteristics (105). Additionally, Erianin, isolated from Dendrobium chrysanthum, inhibits ITGB3-mediated signalling leading to ferroptosis in lung cancer cells, thereby inhibiting their growth, invasion, and metastasis (106). These findings provide compelling pharmacodynamic evidence from animal models for the application of natural products in tumour therapy.

#### 4.3.3 Combination strategies with conventional therapies

Researchers have explored combining TCM and natural products with standard therapies to enhance efficacy and reduce toxicity. A review by Yang et al. (107) highlights that herbal medicines can alleviate the toxicity of conventional therapies while enhancing antitumor effects, serving as auxiliary agents in cancer treatment. Furthermore, addressing the need for improved immunotherapy outcomes, several studies have demonstrated that natural products can modulate immune responses against cancer. Huo et al. (108) systematically reviewed the potential of natural products to target immune cells and remodel the tumor immunosuppressive microenvironment, thereby helping to overcome tumor immune escape. Specifically, natural products have shown potential in animal models to synergize with immune checkpoint inhibitors by regulating immune cells such as T cells and macrophages within the tumor microenvironment (109). These findings offer novel perspectives for the application of TCM and natural products in comprehensive cancer treatment, laying the groundwork for future clinical research.

## 5 Discussion

ITGB3, as a key regulatory molecule in tumour progression, plays a central role across multiple core malignant processes including tumour invasion and metastasis, angiogenesis, immune microenvironment remodelling, and autophagy regulation. By forming heterodimers with distinct α subunits, it activates multi-tiered downstream signalling pathways, establishing a synergistic tumour-promoting network involving cells, the extracellular matrix, and the microenvironment. This unique regulatory mechanism renders ITGB3 an ideal target for tumour diagnosis, prognostic assessment, and targeted therapeutics.

In terms of diagnosis and prognosis, ITGB3 exhibits abnormally high expression in the early stages of multiple tumours. Its expression levels correlate closely with tumour malignancy and patient prognosis, offering a reliable biomarker direction for early tumour screening and disease monitoring. Further optimisation of detection techniques is required to enhance specificity and sensitivity, thereby advancing its clinical translation. Regarding therapeutic approaches, currently developed targeted therapies—including small-molecule inhibitors and monoclonal antibodies—demonstrate certain antitumour effects by directly blocking ITGB3 function or its downstream signalling pathways. Concurrently, traditional Chinese medicine (TCM), leveraging its multi-component, multi-target network regulation advantages, exhibits unique potential in modulating ITGB3 signalling pathways and improving the tumour microenvironment. This offers novel insights for developing integrated treatment strategies combining “precision inhibition with holistic regulation”.

However, the clinical efficacy of these therapies is often limited by acquired resistance. Recent findings highlight three key mechanisms underlying this challenge. First, compensatory integrin upregulation allows tumours to switch dependency, such as from αvβ3 to αvβ5. Second, pathway redundancy enables signalling bypass via other receptor tyrosine kinases (RTKs). Third, microenvironmental shielding, particularly increased ECM stiffness, protects tumor cells from therapeutic agents. Importantly, these resistance mechanisms exhibit significant heterogeneity across tumor types. For example, breast cancer models display distinct resistance profiles compared to lung cancer, largely due to divergent microenvironmental pressures, which aligns with the tissue-specific expression and functional data presented in Section 3.

Despite these advances, several limitations and key future research directions must be addressed. Current limitations include the functional heterogeneity of ITGB3 across different tumor types, the lack of highly specific clinical inhibitors, and unclear mechanisms regarding certain Traditional Chinese Medicine (TCM) compounds. To overcome these challenges, future research should focus on: (1) utilizing multi-omics approaches to map tissue-specific regulatory networks; (2) developing combination strategies, such as pairing ITGB3 inhibitors with immunotherapy, to overcome resistance; and (3) employing modern technologies to elucidate the precise molecular targets of natural products. By concentrating on these key scientific questions through multidisciplinary integration, we can advance the development of ITGB3-targeted diagnostic technologies and therapeutic agents, ultimately realizing ITGB3-guided precision cancer prevention and treatment to improve clinical outcomes.
