# Correction: Integrin β3: structural functions, tumour microenvironment regulatory roles and targeted intervention strategies

**DOI:** 10.3389/fimmu.2026.1847983

**Published:** 2026-04-27

**Authors:** Lianyi Peng, Fuxian Liu, Yue Yang, Liying Zhang, Shuangsheng Huang, Fei Zhao, Caili Li, Jiyu Huang, Zihan Wang, Jianxin He

**Affiliations:** 1 Key Laboratory of Environmental Ecology and Population Health in Northwest Minority Areas, State Ethnic Affairs Commission, Northwest Minzu University, Lanzhou, Gansu, China; 2 Yunnan University of Traditional Chinese Medicine, Kunming, China; 3 Key Laboratory of Ministry of Education for Dunhuang Medicine and Transformation, Gansu University of Chinese Medicine, Lanzhou, Gansu, China

**Keywords:** angiogenesis, integrin β3 (ITGB3), targeted therapy, traditional Chinese medicine, tumour microenvironment

Affiliation “^1^Key Laboratory of Environmental Ecology and Population Health in Northwest Minority Areas, State Ethnic Affairs Commission, Northwest Minzu University, Lanzhou, Gansu, China” was erroneously given as “^1^Key Laboratory of Environmental Ecology and Population Health in Northwest Minority Areas, State Ethnic Affairs Commission, Northwest University for Nationalities, Lanzhou, Gansu, China”.

The original version of this article has been updated.

